# Nonoperative Management of Hepatic Adenomas: A Review

**DOI:** 10.1007/s12029-026-01406-0

**Published:** 2026-02-13

**Authors:** Ian C. Garbarine, Jordan M. Cloyd

**Affiliations:** 1 Department of Surgery, The Ohio State University Wexner Medical Center, Columbus, OH 43210 USA; 2 Division of Surgical Oncology, Department of Surgery, The Ohio State University Wexner Medical Center, Columbus, OH 43210 USA

**Keywords:** Hepatic adenoma, Hepatocellular adenoma, Hepatic adenomatosis, Liver cell adenoma, Liver adenoma, Non-operative management

## Abstract

Hepatic adenomas (HAs) are relatively common benign liver tumors that carry small risks of hemorrhage and malignant transformation, both of which increase with tumor size. While traditionally linked to estrogen-containing oral contraceptives (OCPs), other risk factors including obesity have been associated with HA formation and growth. Treatment strategies depend on the risk of malignant transformation or hemorrhage, tumor size, histological subtype, and patient-specific factors. While traditionally, surgical resection has been the standard recommendation for large, symptomatic, or high-risk tumors, several non-operative strategies are now available to minimize morbidity and optimize outcomes. Active surveillance is recommended for patients with small (< 5 cm), asymptomatic HAs without other risk factors. Loco-regional therapies, including transarterial embolization and radiofrequency ablation, have been used effectively, particularly for small tumors. Mounting evidence suggests that interventions aimed at weight loss, either through lifestyle modifications or bariatric surgery, are efficacious in reducing HA size. Recent data support expanding non-operative management for even large tumors (> 5 cm), as they can respond to lifestyle interventions. Several subgroups of patients warrant special consideration, such as pregnant patients, males, and patients with hepatic adenomatosis. A personalized, multidisciplinary approach remains essential as non-operative strategies continue to expand along with emerging targeted therapies.

## Introduction

Hepatic adenomas (HA), also known as hepatocellular adenomas or liver cell adenomas, are rare, benign neoplasms arising from hepatic epithelial cells. Histologically, HAs are broadly characterized by uniform sheets of hepatocytes with the absence of bile ducts and portal tracts [1]. Landmark studies from the 1970 s determined an association between the use of estrogen-containing oral contraceptives (OCPs) and the development of HAs [2, 3]. At that time, the incidence of HAs in individuals using OCPs was reported to be 3–4 per 100,000 compared with 0.1 per 100,000 for those not using OCPs [2]. The next large-scale epidemiological studies of HA did not occur until the 2010 s, when a Danish study suggested an incidence of 0.07 per 100,000, regardless of OCP use [4]. Despite these recent studies, the incidence of HA is likely underestimated, as most HAs are found incidentally on cross-sectional imaging obtained for another indication [5].

Although linked to OCP use, the introduction of lower estrogen-containing OCPs and alternative contraceptives has not significantly decreased the incidence of HAs, suggesting other drivers of HA formation. Additional recognized risk factors for HA include obesity, metabolic syndrome, elevated androgens (endogenous or exogenous), and genetic disorders, such as glycogen storage disease and mature-onset diabetes of the young-3 (MODY3) [6]. Obese patients have elevated estrogen levels compared to normal-weight individuals, likely due to increased peripheral aromatization of circulating androgens contributing to HA formation in these patients [7]. Additionally, both obesity and HA formation are independently associated with elevated levels of the pro-inflammatory cytokine, interleukin-6 (IL-6) [8].

HAs are classically associated with women of reproductive age, with a reported female-to-male predominance of 9:1, in case series out of Western countries [9]. However, recent studies from China, Japan, and Taiwan have demonstrated a male predominance [9, 10]. These geographic differences may reflect variations in molecular drivers underlying HA formation. In fact, HAs can be divided into six subtypes based on transcription and protein-level findings that correspond to distinct genetic alterations. These subtypes differ in prevalence, risk factors, imaging and histologic features, and prognosis (Table 1). Although usually benign, HAs may rupture or undergo malignant transformation into hepatocellular carcinoma (HCC). The risk of these complications increases with increasing HA size. Adenomas > 5 cm have a reported risk of hemorrhage of approximately 25% and malignant transformation of approximately 4.2% [10]. Exophytic tumors, inflammatory and sonic hedgehog subtypes of HA, high androgen states such as pregnancy, and male gender are associated with increased risk of rupture [6, 14]. Similarly, male gender and tumors harboring a β-catenin mutation are independent risk factors for malignant transformation regardless of HA size [18, 19] (Table 1).

**Table 1:** Six major molecular subtypes of HA and their associated characteristics

*Subtype*	*Prevalence*	*Genetic Alteration*	*Risk Factors*	*Malignant Potential*	*Immunohistochemistry*	*MRI Findings*
HNF-1α (inactivated hepatocyte nuclear factor 1α)	30–35% [11]	Biallelic inactivation of HNF1A tumor suppressor gene [11]	Obesity, MODY type 3 [6]	Lowest of any subtype [12]	Downregulation of LFABP, β-catenin, and glutamine synthase [11]	Decreased signal intensity at T1-weighted opposed-phase (reflects increased intracellular fat [13]
Inflammatory (IHA)	35–40% [14]	Activating mutation of IL-6/JAK/STAT pathway [6, 11]	Obesity (strong association), alcohol use, glycogen storage disorders [13]	Highest risk of hemorrhage [1]	Strong expression of serum amyloid A and C-reactive protein [15]	Marked T2 hyperintensity; persistent enhancement during portal venous phase [13]
β-catenin-activated (βHA)	15–20% [12]	Missense mutation in CTNNB1 at exon 3 or exon 7/8 [6]	Glycogen storage disease, vascular disease, androgen exposure [6, 12]; male sex predominance [4, 12]	Highest malignant potential (especially exon 3 mutations) [16]	Strong, homogeneous cytoplasmic glutamine synthetase expression; nuclear β-catenin positivity in exon 3 mutations only [17]	Most exon 3 mutations show iso- or hyperintense signal intensity [13]
Sonic Hedgehog	4% [11]	Activating mutations of sonic hedgehog pathway [11]	Obesity, estrogen-containing OCP use [6]; hepatic steatosis	Elevated risk: resection generally preferred [11]	Hypervascularity [11, 13, 14]	No definitive imaging characteristics [13]
Unclassified (UHA)	Remaining cases	Diagnosis of exclusion [6, 11, 13]				

*MODY* Mature onset diabetes of the young, *LFABP* Liver-type Fatty Acid-Binding Protein, *IL-6* interleukin-6, *JAK* Janus kinase, *STAT* Signal Transducer and Activator of Transcription, *OCP* Oral contraceptive pills

## Surgical Resection

While the majority of HAs will not require intervention, surgical resection should be considered for patients at higher risk of rupture, malignant transformation, disabling symptoms, or inability to rule out HCC. Specifically, traditional indications for resection include size > 5 cm, prior rupture, male sex, rising aminotransferase levels, underlying genetic metabolic diseases, or tumors that carry a β-catenin mutation [18–20]. Given advances in perioperative medicine and surgical technique, elective liver surgery can now be performed at experienced centers with increasingly low morbidity and mortality [21]. In addition, hepatic resection is increasingly performed in a minimally invasive fashion [22]. Nevertheless, patients can still experience complications [23] and/or diminished long-term quality of life after hepatic resection for HA [24]. Therefore, the purpose of this review is to provide an overview of HA management options, highlighting key aspects of non-surgical interventions.

## Methods

A comprehensive review of the English literature was performed utilizing PubMed, Embase, Cochrane Library, and Scopus databases. The following search terms were used: l*iver cell adenoma*,* liver adenoma*,* adenoma*,* hepatic adenoma*,* hepatocellular adenoma*, and *adenomatosis*. The initial search was completed on September 30, 2025. References from relevant articles were reviewed to identify additional publications. An expert review of the relevant literature was performed, and the most relevant publications were included.

## Active Surveillance and Cessation of OCPs

Both the American College of Gastroenterology (ACG) and the European Association for the Study of the Liver (EASL) have published guidelines for the workup and care of patients with HAs (Table 2) [20, 25]. Both guidelines suggest a broad workup for patients with incidentally detected liver lesions, including obtaining baseline laboratory studies (liver function tests, circulating tumor markers, and viral hepatitis panel) and contrast-enhanced imaging. In patients with cirrhosis, any new lesion should be presumed HCC until conclusive information suggests an alternative etiology. Both ACG and EASL guidelines suggest reserving biopsies for cases in which a hepatic adenoma has an atypical appearance on imaging or concerning findings for malignant transformation. Similarly, both societies suggest multidisciplinary discussion for diagnostic dilemmas. Recent advances in understanding the genetic makeup of HA have enabled the identification of distinct subtypes, leading to improved prognoses and risk stratification. Enhanced imaging techniques have enabled the pairing of imaging findings from high-quality MRI with subtypes [9, 12].

**Table 2:** Overview of EASL and ACG guidelines for hepatic adenomas

*Category*	*EASL (2016)* [25]	*ACG (2024)* [20]
Initial Imaging	MRI preferred as first-line assessment	Multiphasic MRI preferred
Lifestyle Modifications	Discontinue OCPs and weight loss advised	Discontinue OCPs/hormone IUDs and weight loss for overweight/obese
Resection in Men	Recommended irrespective of size	Implied high-risk approach
β-catenin Mutated HCA	Resection recommended irrespective of size	High-risk approach
Women with HCA < 5 cm - Initial Management	6-month observation after lifestyle changes	Discontinue hormones and weight loss with surveillance imaging
Women with HCA < 5 cm - Surveillance	Reassess at 1 year, then annual imaging	CT/MRI every 6–12 months for 2 years, then annually
Women with HCA ≥ 5 cm	Resection after 6 months of lifestyle changes if still ≥ 5 cm	Resection typically indicated
Long-term Surveillance	Annual imaging can extend to every 2 years if stable after 5 years	Annual imaging after initial 2-year period
Alternative to Surgery	Not significantly featured	Embolization or ablation suggested when surgery not feasible

Active surveillance is recommended for patients who do not meet criteria for surgical resection. In the absence of worrisome characteristics, close follow-up with multiphasic contrast-enhanced CT or MRI is recommended every six months for the first two years, then annually. Initial treatment for patients with HAs who take exogenous steroid hormones (e.g., estrogen, androgens), such as for the treatment of aplastic anemia, gender-affirming care, hyperandrogenism, or contraception, consists of immediate cessation of these agents. HA size reduction after stopping the offending agent has been well described and can often obviate surgical intervention [20, 25, 26]. Repeat imaging and clinical follow-up should be pursued in six months. Both ACG and EASL guidelines suggest that surgical resection should be considered if a lesion increases by ≥ 20% in size during this timeframe [20, 25]. The timing of resection after cessation of OCP, however, is debated, with evidence from several case reports and series suggesting that for selected patients, an extended observation period may be appropriate [27–30]. For example, a 2017 retrospective study by Klomepner et al., which included 194 patients with HA greater than 5 cm over 16 years, found that the median time for adenoma to regress to less than 5 cm was approximately 21.3 months (IQR 20–32 months) [28]. Within the follow-up period, no complications were reported. Similarly, a 2019 study by Haring et al. of 78 patients with HAs found that approximately 98% of HA remained stable or regressed after cessation of OCP, with a median follow-up of 1.3 years (IQR 0.6–2.6 years) [29]. Both studies reported no HA-induced complications and suggested an increased rate of regression associated with larger HA [28, 29]. These findings support extended observation of large HAs if regression is reserved after OCP cessation.

## Weight Loss, Metabolic Control, and Lifestyle Interventions

**Fig. 1:**
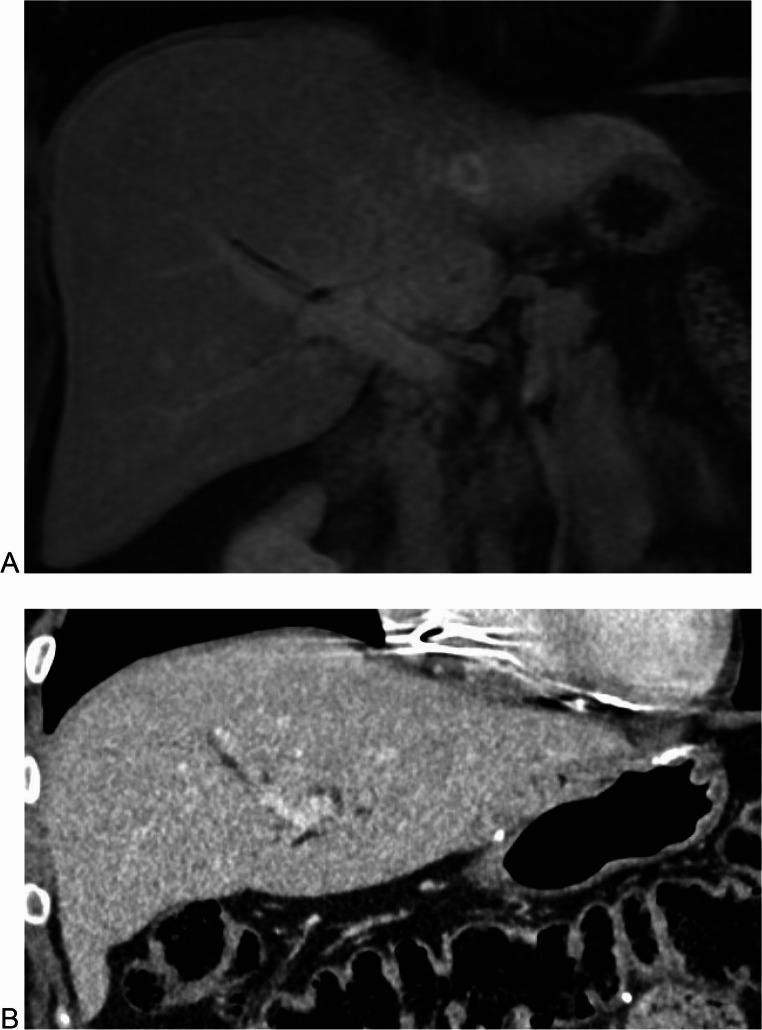
Representative cross-sectional images from a patient with HA pre- and post-bariatric surgery (Roux-en-Y gastric bypass). (**A**) MRI showing hepatic adenoma in segment II (1.8 cm x 1.4 cm) (**B**) CT captured during post-operative follow-up without evidence of HA in segment II. Patient’s BMI pre-op was 38.03 kg/m^2^ and at time of follow-up imaging was 23.73 kg/m^2^

Given the association of obesity with HA formation, interventions aimed at weight loss can be effective for patients with HAs not using OCPs [20, 25, 31, 32] (Fig. 1). In fact, ACG guidelines strongly recommend weight loss for overweight or obese patients with HAs as part of an initial management strategy [20]. Beegle et al. reported on a case series of eight individuals with HAs in the setting of glycogen storage disease who had significant regression after strict metabolic control [31]. Dokmak et al. reported two cases of female patients with HAs greater than 5 cm without recent OCP use. These two patients had significant regression of their HAs (> 50%) to sizes < 5 cm after significant weight loss (average of 31% reduction in BMI) obtained through diet and exercise [32].

The recent widespread use of glucagon-like peptide-1 (GLP-1) receptor agonists for weight loss has transformed the treatment of obesity. These medications function by stimulating endogenous insulin release, while also inhibiting gastric emptying and endogenous glucagon release [33]. Several large clinical trials have demonstrated that these medications can result in a mean weight loss of more than 10% [34, 35]. Additionally, several studies have shown an improvement in transaminitis and even resolution of non-alcoholic steatohepatitis (NASH) with the use of these agents [24, 26]. Considering the association between weight loss and reduced HA size in obese patients, GLP-1 receptor agonists may be attractive therapeutic options for treating HA, with potential for hepatoprotective effects. However, to date, no studies have investigated the role of these medications in treating HAs.

Bariatric surgery, most commonly through gastric bypass or sleeve gastrectomy, has been shown to cause significant and sustained weight loss more commonly than conservative efforts, such as diet and exercise. In fact, bariatric surgery remains the most effective targeted obesity treatment, offering the highest rates of sustained weight loss and is associated with the resolution of obesity-related comorbidities in many patients [36, 37]. Several small case series and systematic reviews have shown an association between a reduction in the size of HA, and the percentage of excess weight loss after bariatric surgery [38–40]. These findings, along with the lower rates of weight regain after bariatric surgery compared to more conservative weight loss interventions, suggest that bariatric surgery may be considered for the initial management of HA, especially for patients who are unable to sustain weight loss with conservative measures, or for HA > 5 cm without other high-risk features.

## Local-regional Therapies

Local-regional therapies, including transarterial embolization (TAE) and radiofrequency or microwave ablation (RFA/MWA), enable control of HAs with less invasive measures than surgical resection. While TAE has shown significant benefits in controlling hemorrhage from ruptured HAs in hemodynamically stable patients, the use of TAE as well as RFA/MWA, in the elective setting has increased over recent years [41–43]. These modalities are particularly beneficial for patients with multiple adenomas, those unable to undergo surgery (e.g., comorbidity or pregnancy), or with tumors that are not amenable to resection (e.g., due to anatomic location) [18, 44, 45]. In fact, EASL guidelines do recommend considering local-regional therapies for patients who are poor surgical candidates [25].

Several large multinational case series have suggested that TAE is safe for the elective treatment of HAs, and reported perioperative morbidity ranging from 7.9% to 11.9%, with serious morbidity of 5.3% to 8.5% [42, 46]. Rosmalen et al. reported on 56 patients who underwent elective TAE and showed a significant average decrease in HA size from 70 mm to 51 mm (*p* < 0.001). Among patients with HAs > 5 cm, 75% had tumor regression < 5 cm. However, approximately one-third of patients initially treated with TAE required surgical resection [46]. In a meta-analysis of 151 patients, 45% were able to avoid further surgical resection and 75% of patients experienced tumor regression after TAE. For those patients undergoing elective TAE (49 patients), 84% did not require further surgical intervention. This study reported an overall and major complication rate of 7.9% and 5.3% respectively [42]. No tumor growth or malignant transformations were noted. Complications of TAE include acute renal injury, intrahepatic cysts, and postembolization syndrome [18, 42].

The use of transarterial chemoembolization (TACE) for HA has not been well studied, although small case series have suggested efficacy in the treatment of HAs, including hepatic adenomatosis [47, 48]. Although HAs have malignant potential, adding chemotherapeutic agents exposes patients to the risk of chemotherapy-related adverse effects, without significant benefits over bland embolization [49].

Thermal ablation has been used extensively and successfully to treat small (< 3 cm) localized primary and secondary liver cancers [50, 51]. However, the role of thermal ablation in HA is less well studied. Van Vladder et al. described a series of 18 patients (HAs 1–14 cm) who underwent percutaneous (*n* = 28) or open (*n* = 4) RFA ablation using a single probe. Patients had a median size of 3 cm. The primary technical efficacy rate (defined as successful ablation at the index ablation) was 57.8%. In contrast, Laimer et al. reported a single-institutional experience with RFA comprising 14 patients, with a primary technical efficacy rate of 97.4% [52]. Mironov et al. reported a single-institution retrospective cohort study consisting of 36 patients with 58 tumors [53]. Median tumor size was 2.1 cm, and single-probe RFA was performed under ultrasound guidance. The authors report a primary technique efficacy of 88%, a complication rate of 4.5%, and no evidence of malignant transformation during the follow-up period. These data suggest that thermal ablation is safe and effective, particularly for those HAs < 3 cm. Nevertheless, because most small, solitary, asymptomatic HAs do not require intervention, the role of RFA/MWA in patients with HAs is limited.

## Special Populations

### Pregnancy

Pregnancy is associated with elevated levels of estrogen and androgens, which can promote HA growth and lead to rupture [54]. Traditionally, due to the higher mortality associated with ruptured HA during pregnancy, patients with HA were advised to avoid pregnancy or undergo HA resection beforehand [44]. However, recent data have suggested that a more conservative approach, especially for small HAs, may be feasible [45]. A systematic review by Haring et al. comprising 90 patients with 99 pregnancies, reported that 53.4% of HAs remained stable, 15.1% regressed, and 31.5% progressed. Eight patients (8.1%) experienced HA bleeding during pregnancy, two during labor (2.0%), and five (5.1%) postpartum, although all these patients had HA > 6.5 cm [55]. These findings suggest that the presence of small, asymptomatic HAs without other high-risk features should not automatically prompt the need for surgical resection prior to or during early pregnancy [56]. In these cases, observation with ultrasounds every 6–12 weeks has been proposed [57]. A prospective study by Gaspersz et al. followed 48 patients with imaging-confirmed HA < 5 cm during 51 pregnancies. Patients were followed with liver ultrasounds at 14, 20, 26, 32, 38 weeks of gestation and 6–12 weeks postpartum. HA growth > 20% was seen in 25% of participants, with a median increase of 1.4 cm. 53% of patients had stable lesion size, 22% had lesions that regressed. One patient experienced a 2.7 cm growth and was successfully treated with TAE. These data support monitoring HA with serial ultrasounds during pregnancy [54]. Those HAs increasing in size during pregnancy, are symptomatic, or carry other risk factors for rupture, should still be considered for intervention. When indicated, societal guidelines suggest resection before 24 weeks of gestation or local-regional treatment, such as TAE, after 24 weeks [25].

### Male Gender

Approximately 10–20% of HAs occur in males, with a higher proportion reported in Eastern compared to Western countries [9, 58–60]. Risk factors more common in males include the use of anabolic steroids and glycogen storage disease [58]. Rising rates of obesity and metabolic syndrome likely underlie the increasing incidence in male patients over the recent decades.

Of note, HAs in male patients have higher rates, reported up to approximately 50%, of malignant transformation to HCC, presumed to be driven by a higher proportion of underlying β-catenin exon 3 mutations [4, 12, 60]. Due to this risk, EASL and ACG recommend surgical resection in all males regardless of HA size. However, recent work has suggested that a more nuanced approach based on the subtype of HA may be more appropriate. González et al. analyzed the molecular subtypes of HAs in 27 male patients in the U.S., finding that inflammatory-HCA was the most common subtype (37%), followed by β-catenin-activated HAs (b-HA; 14.8%). Of the patients studied, only those with b-HAs and those described as unclassified HA were associated with concomitant HCC [61]. These studies report a lower proportion of β-catenin than previously reported, suggesting that the risk of malignant transformation observed in males is due to other risk factors, such as underlying liver disease, or to misdiagnosis of already transformed HCC as β-HA.

A recent single-center study by Jain et al. of seven male patients (86% were obese) showed a high percentage (57.1%) of patients harbored an inflammatory subtype HA. No patients had a subtype with a high risk of malignant transformation [59]. These data suggest that risk stratification based on HA subtype and underlying risk factors, a low-risk subgroup of males, may warrant non-surgical approaches initially, particularly HAs in obese males [62]. For male patients with HAs harboring β-catenin mutations, underlying risk factors, or an uncertain HA vs. HCC, upfront resection remains warranted. Mauro and Forner have proposed a schema for the management of male HAs. If an uncomplicated HA (e.g., no exophytic features, no evidence of hemorrhage) is identified on cross-sectional imaging, a biopsy of the lesion is subsequently performed. If the biopsy is negative for β-catenin mutations and there is an absence of patient-level risk factors for HCC (e.g., MAFLD, viral hepatitis infection, cirrhosis), the patient can be observed with serial imaging along with lifestyle changes (e.g., weight loss, cessation of offending agents) with cross-sectional imaging every six months. If the tumor grows, shows an appreciable change on imaging, remains > 5 cm despite lifestyle interventions, or becomes symptomatic, then resection is pursued [63].

### Hepatic Adenomatosis

Hepatic adenomatosis is a variant of HA, traditionally defined as ≥ 10 HAs, though some authors suggest ≥ 4 adenomas as the threshold [64]. In addition to associations with glycogen storage disease, obesity, and metabolic syndrome, hepatic adenomatosis is associated with hepatic steatosis and vascular abnormalities, such as portal vein agenesis, unlike isolated HAs. Reported complication rates vary considerably, with hemorrhage risk ranging from comparable to isolated HA to more than two-fold higher (25–62.5.5%), and malignant transformation risk similarly estimated at 4–10% [20, 56, 65]. This variability likely reflects heterogeneity in molecular subtype distribution across small studies of this rare condition, given that risk for both complications correlates more strongly with molecular subtype than with number of lesions [66]. Barbier et al. described the largest cohort of hepatic adenomatosis, comprising 40 cases at a single center. This cohort had a female predominance (90%), with most patients (80%) presenting with symptomatic (e.g., bleeding, persistent abdominal pain), large nodules > 5 cm. On pathologic assessment, HAs shared the same molecular classification in 87%. In this case series, malignant transformation to HCC occurred in 2.5% and hemorrhage occurred in 15% although overall complications were not more frequent than in isolated HA. Long-term follow-up of this cohort showed either the development of new lesions or the growth of preexisting lesions in 23% of patients [66].

EASL and ACG guidelines suggest that intervention for hepatic adenomatosis be based on the size of the largest lesion or the presence of risk factors [56], although generally resection is not feasible given the number and bilobar location of the tumors. Lifestyle interventions, including OCP cessation and weight loss, led to regression in approximately 33% of patients [65]. For patients who are unable to undergo resection, local-regional interventions such as TAE are often recommended after cessation of OCPs. While there have not been large, prospective studies on local-regional control of hepatic adenomatosis, several case studies have shown improvement in multiple lesions after TAE or RFA [52, 67]. For instance, van Vledder et al. described 10 patients with multiple tumors (median of two tumors, range of one to 12 tumors), with three patients having 10 or more lesions and two patients undergoing successful ablation of 75–90% of lesions. One of three patients had follow-up and was noted to have regression of ablated lesions. For patients with hepatic adenomatosis, liver transplantation could be considered when refractory to other nonoperative measures and/or concern for malignancy transformation, especially in the setting of underlying liver disease such as glycogen storage disease [64].

## Emerging Targeted Therapies

Several medical therapies have been studied in the treatment of HA, especially in the setting of hepatic adenomatosis (Table 3). These drugs target recurrent activating mutations such as in Fyn-related Src kinase (FRK), STAT3, interleukin-6 signal transducer (*IL6ST*), and Janus kinase (JAK)1 pathways [72]. Several pre-clinical studies have demonstrated that inflammatory HAs (IHAs) harboring genetic mutations in IL6ST affecting protein gp130, which leads to constitutive activation of STAT3 (present in approximately 70% of IHAs), respond to JAK/STAT inhibition with agents such as ruxolitinib, a JAK1/JAK2-selective inhibitor [68, 69]. Tofacitinib, another small molecule JAK inhibitor, has demonstrated similar effects on IL-6-mediated signaling in hepatocytes and may represent an alternative therapeutic option, although it has not been directly studied in HA [78].

**Table 3:** Emerging medical therapies for hepatic adenomas

Target	Mechanism of Action	HA Subtype	Agents
JAK1/JAK2 [68, 69]	Selective JAK inhibition; blocks constitutive STAT3 activation in IHAs with gp130 mutations	IHA	**Ruxolitinib**, tofacitinib
IL-6/IL-6 Receptor [70, 71]	Monoclonal antibody blockade of IL-6 signaling; inhibits downstream JAK/STAT activation	IHA	Tocilizumab, siltuximab
FRK/STAT3 [72]	Non-specific Src kinase inhibition; blocks oncogenic FRK signaling and STAT3 activation	HAs with FRK mutations	**Dasatinib**
GLI1/GLI2 [73, 74]	Direct or indirect GLI inhibition; blocks sonic hedgehog pathway transcription	shHA	**GANT61**, **MEK/Raf inhibitors**
PPARα [75, 76]	PPARα agonism; suppresses IL-6-mediated acute phase response and hepatic inflammation	IHA	**Fenofibrate**
GLP-1 Receptor [34, 35, 77]	GLP-1R agonism; promotes weight loss, reduces hepatic steatosis, decreases systemic inflammation	Obesity-associated HA, IHA	Semaglutide, liraglutide, tirzepatide

Agents in **bold** have preclinical or clinical evidence in hepatic adenomas

*HA* hepatic adenoma, *IHA* inflammatory hepatic adenoma, *shHA* sonic hedgehog hepatic adenoma, *JAK* Janus kinase, *STAT* signal transducer and activator of transcription, *FRK* Fyn-related Src kinase, *GLI* glioma-associated oncogene, *PPARα* peroxisome proliferator-activated receptor alpha, *IL-6* interleukin-6, *IL-6R* interleukin-6 receptor, *GLP-1R* glucagon-like peptide-1 receptor

Likewise, dasatinib, a non-specific Src kinase inhibitor may be effective in the treatment of HAs through blockade of STAT3 and FRK. In fact, in one preclinical study, dasatinib administration significantly reduced the oncogenic activity of FRK mutant cells lines. Additionally, mice with orthotopically transferred constitutively active FAK mutant tumor cells had improved tumor control compared to controls [72]. A subset of HAs has sonic hedgehog activating mutations (shHA). These tumors can be targeted with small molecule inhibitors targeting GLI1 either directly, such as with GANT61 or indirectly via MEK and Raf inhibitors [73, 74].

Fenofibrate, a peroxisome proliferator-activated receptor (PPAR) alpha agonist, has been shown to reduce IL-6-mediated acute inflammation [75]. In a case report, Poupon et al. described a 52-year-old obese woman with multiple biopsy-proven IHA lesions who declined surgical intervention. The patient experienced regression of her lesions after initiation of fenofibrate therapy, suggesting a potential role for fenofibrate in treating IHAs [76]. Monoclonal antibodies targeting IL-6 (siltuximab) or IL-6 receptors (tocilizumab) represent additional possible approaches to target IL-6 driven inflammation in IHAs although these agents have not been studied in the context of HAs [70, 71]. Genomic-based medical therapy is promising, but further preclinical and prospective clinical trials are needed.

## Conclusions 

Hepatic adenomas are rare, benign tumors of the liver, but carry a risk of hemorrhage and malignant transformation into hepatocellular carcinoma that increases with size. While surgical resection has traditionally been considered the definitive option for at-risk HAs, recent evidence suggests that conservative measures (e.g., cessation of estrogen-containing OCPs, weight loss, etc.) and active surveillance should be the initial step for most patients. Furthermore, non-operative interventions can be useful for patients at prohibitively high risk for surgery or who are not candidates, given the number or location of HAs. These strategies can minimize the use of complex liver surgery in low-risk HAs, reserving resection for those with the highest risk of malignant transformation, rupture, or other complications. Continued refinement of HA subtypes and patient-level risk factors will improve risk stratification and prognostication. Ongoing research is not only investigating the pathogenesis of HAs but also the role of local-regional and targeted medical therapies.

## Data Availability

No datasets were generated or analysed during the current study.
